# CROSS-CULTURE ADAPTATION AND VALIDATION OF THE CHINESE VERSION OF MANCHESTER COPD FATIGUE SCALE (MCFS)

**DOI:** 10.1093/geroni/igae098.1452

**Published:** 2024-12-31

**Authors:** Haihong Zhang, Chongmei Huang, Minhui Liu

**Affiliations:** Ningxia Medical University, Yinchuan, Ningxia, China (People’s Republic); Ningxia Medical University, Yinchuan, Ningxia, China (People’s Republic); Ningxia Medical University, Yinchuan, Ningxia, China

## Abstract

Fatigue is an aching, complex, and multifaceted sensation that is the second most prominent and disabling symptom of COPD, approximately 50% of COPD patients suffer from severe fatigue. Various scales or questionnaires has been developed to measure fatigue in COPD patients. The Manchester COPD fatigue scale (MCFS) has been proved to be a robust, simple, reliable, and valid scale in the American population with COPD. As a disease-specific measurement, MCFS can precisely identify the features of fatigue among COPD patients in the physical, cognitive, and psychosocial domains. The Brislin’s translation model was used to for cross-cultural adaptation. Internal consistency, test-retest reliability, constructive validity, convergent validity, and criterion validity were used to test the reliability and validity of MCFS. Diagnostic accuracy was used to determine the optimal cut-off point. Overall, a convenience sampling of 371 patients completed the fatigue scale. The results showed that the scale has acceptable constructive validity (CFI = 0.877, IFI = 0.878, SRMR = 0.027), excellent internal consistency (Cronbach’s alpha = 0.97) and test-retest reliability (Cronbach’s alpha=0.93). MCFS presented good convergent validity. Using the BFI fatigue scale as a reference criterion, the areas under the curve were 0.941 and 0.900, respectively. The optimal cut-off points for mild, moderate, and severe fatigue were ≤16, >16 and ≤29.5, and >29.5 (sensitivity: 94.7% and 82.6%; specificity: 80.8% and 86.0%, respectively). The Chinese version of MCFS could be used to measure fatigue among Chinese patients with COPD.

